# Immune response development after vaccination of 1-day-old naïve pigs with a Porcine Reproductive and Respiratory Syndrome 1-based modified live virus vaccine

**DOI:** 10.1186/s40813-018-0112-7

**Published:** 2019-02-02

**Authors:** Monica Balasch, Maria Fort, Lucas P. Taylor, Ivan Díaz, Enric Mateu, Jay G. Calvert

**Affiliations:** 1 Zoetis Manufacturing & Research Spain S.L., Ctra. Camprodon s/n, Finca La Riba, 17813 Vall de Bianya, Girona Spain; 2 Zoetis Inc, 333 Portage St, Kalamazoo, MI 49007 USA; 3 UAB, Centre de Recerca en Sanitat Animal (CReSA, IRTA-UAB), Campus de la Universitat Autònoma de Barcelona, 08193 Bellaterra, Barcelona Spain; 4 Departament de Sanitat i Anatomia Animals, Facultat de Veterinària, UAB, 08193 Bellaterra, Barcelona Spain; 5 IRTA, Centre de Recerca en Sanitat Animal (CReSA, IRTA-UAB), Campus de la Universitat Autònoma de Barcelona, 08193 Bellaterra, Barcelona Spain

**Keywords:** *Porcine reproductive and respiratory syndrome virus*, Cell-mediated immunity, Serum Neutralising antibodies, Modified live virus vaccine

## Abstract

**Background:**

The development of the innate and adaptive immune responses to *Porcine reproductive and respiratory syndrome virus* (PRRSV) after vaccination of 1 day-old pigs with a PRRSV-1 based modified live virus (MLV) vaccine by intramuscular (IM) and intranasal (IN) routes was characterised, before and after challenge with a heterologous PRRSV-1 isolate at 18 weeks post-vaccination. Twenty-five PRRSV-seronegative piglets were used. At 1 day of age, pigs were administered with a single dose of vaccine via the IM (*n* = 10) or the IN route (*n* = 10). Control group (*n* = 5) received saline solution. After vaccination, pigs were bled at days 3, 7, 28, 56, 83, 113 and 125. Levels of cytokines IL-10, IL-8, IFN-α (measured by ELISA tests of serum), TNF-α and IFN-γ (measured by ELISA and ELISPOT, respectively, from stimulated peripheral blood mononuclear cells), and serum neutralising antibodies (NA) to the vaccine strain, were measured.

**Results:**

The induction of IL-10 was rare, indicating that IL-10 mediated immunomodulation/immune dysfunction was not a feature of this vaccine or of the challenge virus. IL-8 was detected in only two pigs following vaccination, but in the majority of pigs after challenge, indicating that their ability to produce an innate immune response was not impaired. TNF-α was not detected in any vaccinated pigs until day 83. After challenge, only a minority of pigs produced TNF-α. IFN-α was detected in all vaccinated pigs following vaccination, indicating the potential for development of an effective Th1 adaptive immune response. IFN-γ-secreting cells were detected in all vaccinated pigs after vaccination. NA to the vaccine strain were first detected at day 56 in pigs vaccinated by both routes, and remained at similar levels until challenge. After challenge, a boost in NA was observed. The efficacy of the vaccine was demonstrated by reduction of viraemia and nasal shedding after challenge.

**Conclusions:**

The administration of a PRRSV-1 based MLV vaccine to 1 day-old piglets was able to induce an immune response characterised by: (1) undetectable or low levels of IL-10, IL-8 and TNF-α, (2) an increase in IFN-α expression within the first seven days, (3) a gradual increase in the number of antigen-specific IFN-γ-secreting cells, and (4) induction of detectable NA. After challenge with a heterologous strain, there was a rapid boost in NA titres, indicating a priming effect of the vaccine.

## Background

The development of an adaptive immune response to *Porcine reproductive and respiratory syndrome virus* (PRRSV) depends on both humoral and cellular components [1]. The innate immune system is the first mechanism of defence to prevent viral invasion and replication and influences the adaptive response of the immune system. Adequate early activation of the innate immune system is critical to initiate generation of protective adaptive immunity to achieve complete viral clearance [2]. However, PRRSV induces a slow and weak innate response [1]. The quantities of important cytokines secreted in pigs infected by PRRSV, like IFN-α or IFN-γ, appeared to be significantly lower than in pigs infected with *Influenza A virus* or *Porcine respiratory coronavirus* [3–5]. The development of neutralising antibodies (NA) is also known to occur later in the course of the infection although non-neutralising antibodies appear by 7–14 days post-inoculation [6]. In that way, both humoral and cell-mediated specific immunity are delayed, compromising clearance of the virus [7].

Serum NA also play an important role in the protection of animals against clinical disease. Passive transfer of NA to pregnant sows (titres 1/16) can protect them against reproductive failure by blocking transplacental infection [8]. Using the same antibody transfer system, a titre of 1/8 or higher protected piglets against the development of viraemia, with sterilising immunity being obtained at titres of 1/32 [9]. These results suggest that a vaccine capable of inducing NA titres of 1/32 against a given PRRSV strain should prevent the clinical disease produced by it and could be an important tool in the control of PRRSV [10]. It has to be taken into account, however, that these studies used a homologous challenge model. Despite the significant role that NAs seem to play in protection, their effectiveness might be limited against heterologous isolates, as demonstrated by the limited ability of PRRSV hyperimmune sera to effectively neutralise a variety of heterologous strains [11].

The nature of the protection induced by PRRSV MLV vaccines against heterologous challenges is controversial. Although it is widely accepted that heterologous protection is rather limited and strain dependent [12], contrasting models of the immune response have been proposed for different PRRSV strains: one based on the development of NA with low IFN-γ responses, the other based on effective IFN-γ responses with a poor development of NA [12].

Taken together, it is highly recommended to define the profile of humoral and cell-mediated immunity induced by a given MLV vaccine in order to predict its ability to provide strong protection in front of challenge with heterologous strains.

The ability of the immune system to fight against infectious agents is conditioned by a sufficient degree of functional maturation of the immune system [13]. It has been demonstrated that the ability of monocytes and neutrophils from young pigs to produce pro-inflammatory cytokines is reduced compared to those from adult pigs: the most pronounced changes in cytokine production occurred at weaning [13]. It should therefore be expected that the ability of the immune system of a pre-weaning pig to respond to a MLV vaccine could be reduced relative to older animals.

The objective of this study was to define the profile of development of innate and adaptive immunity to PRRSV after vaccination of 1-day-old pigs with a PRRSV-1 based MLV vaccine (Suvaxyn PRRS MLV), and after challenge with a virulent heterologous field isolate of PRRSV-1. One day old piglets were selected as they represent the “worse-case scenario” (youngest pigs) allowed by the indications of the vaccine used. Two routes of vaccine administration, intramuscular (IM) and intranasal (IN), were evaluated. Challenge was delayed until 18 weeks after vaccination to ensure satisfactory duration of immunity. Innate immunity was measured by evaluating the level of anti-inflammatory and pro-inflammatory cytokines (IL-10, IL-8, TNF-α and IFN-α), humoral immunity by measuring the development of NA, and cell-mediated immunity by evaluating the development of IFN-γ- secreting cells.

## Methods

### Experimental design

Twenty-five piglets born from PRRSV-seronegative sows were used, thus the potential impact of maternally derived antibodies was not assessed in this study. The animals were allocated to treatments following a completely randomised design. At 1 day of age, two groups of 10 pigs were administered a single 2 mL dose of vaccine via the IM (T02) or the IN (T03) route. Five pigs from the control group (T01) received 2 mL intramuscular and 2 mL intranasal of saline solution. After vaccination, pigs were bled at days 3, 7, 28, 56, 83, 113 and 125. Animals were housed by treatment with one pen per treatment in the vaccination phase. Prior to challenge, animals were re-housed and comingled in one room. At 18 weeks post-vaccination (126 days), pigs were challenged IN with the PRRSV-1 isolate Olot/91. Levels of cytokines interleukin-10 (IL-10), interleukin-8 (IL-8), interferon-alpha (IFN-α) and tumor necrosis factor-alpha (TNF-α), number of interferon-gamma (IFN-γ) secreting cells, and levels of NA to the vaccine strain, were measured at different time points after vaccination, and 6 days after challenge. Also, PRRSV viraemia and nasal shedding were determined by RT-qPCR 1 day prior to challenge and at days 3, 6, 8 and 10 after challenge. Ten days post-challenge, pigs were euthanised.

Throughout the experimental period the animals were monitored daily for clinical observations.

### Vaccination

Newborn piglets were used at 24 ± 12 h of age. The commercial vaccine Suvaxyn PRRS MLV contains a derivative of the PRRSV-1 field isolate 96 V198, which has been attenuated by serial passage on BHK21 cells engineered to express the porcine CD163 PRRS receptor. This vaccine was used, at minimum immunising dose (2.2 log_10_ CCID_50_/dose), for T02 and T03 groups. At day 0, piglets in T02 were injected IM in the right side of the neck. Piglets in T03 were administered IN, delivering 1.0 mL in each nostril. Piglets in T01 received saline solution (2 mL IM and 2 mL IN).

### Challenge

At 18 weeks post-vaccination (126 days), pigs were challenged IN with the PRRSV-1 isolate Olot/91 [14], at a dose of 10^5.8^ CCID_50_/pig. The challenge virus is passaged only on primary porcine alveolar macrophages in order to ensure retention of virulence. The degree of ORF7 and ORF5 nucleotide identity between the vaccine virus and the challenge virus is 93.0 and 89.6%, respectively. Although both viruses belong to subtype 1 of PRRSV-1, they differ significantly in sequence.

### Sampling

All pigs were bled before vaccination. After vaccination, pigs were bled at days 3, 7, 28, 56, 83, 113 and 125. After challenge (day 126), pigs were bled and nasal swabs were taken at days 3, 6, 8 and 10 (study days 129, 132, 134 and 136).

Blood samples collected before vaccination (day 0) and before challenge (day 125) were tested by PRRSV ELISA (PRRS × 3, IDEXX) following manufacturer’s instructions.

Blood was collected in appropriate tubes to obtain serum (PRRSV NA assay, PRRSV RT-qPCR and cytokine ELISA tests for IL-10, IL-8 and IFN-α), and whole blood in heparinised tubes (IFN-γ ELISPOT and TNF-α ELISA). Nasal swabs were placed in 1 mL of PBS.

### Cytokine analysis

#### Sera

Cytokines IL-8 and IL-10 in sera were measured by ELISA using commercial pairs of monoclonal antibodies (mAb) and according to the manufacturer’s instructions (Porcine IL-8 and IL-10 ELISA Kits, Thermo Fisher Scientific). The cut-off point of each ELISA was calculated as the mean + 3SD OD (optical density) of negative controls. Cytokine concentrations were calculated by using the linear regression formula from ODs of the cytokine standards provided by the manufacturer. Results were expressed as pg/mL. IFN-α was measured as previously reported [15] at days 3 and 7. Briefly, plates were coated with anti-pig IFN-α (mAb K9, R&D systems) at 1.1 Ug/mL; 50 μl of each sample and biotinylated pig IFN-α (mAb F17, R&D systems) were added. Positive reactions were revealed using Streptavidin-Horseradish (Thermo Fisher Scientific) and soluble TMB (Merck Millipore). IFN-α concentrations were calculated according to the ODs obtained from the serial dilutions of the recombinant porcine IFN-α protein (R&D systems), from 250 to 4 U/mL.

#### Peripheral blood mononuclear cell (PBMC) cultures

PBMC cultures were obtained from heparinised blood samples by density-gradient centrifugation with Histopaque 1077 (Sigma). To measure TNF-α responses, PBMC were seeded at a density of 1 × 10^6^ cells per well in 96-well plates and were mock-stimulated or stimulated with either PRRSV 96 V198 (vaccine strain) at a multiplicity of infection (moi) of 0.1, or 10 mg/ml of PHA. After 24 h incubation, cell culture supernatants were collected and frozen at − 80 °C until used. A TNF-α commercial ELISA test (Porcine TNF-α ELISA Kit, Thermo Fisher Scientific) was performed as above. To calculate PRRSV-specific quantity of TNF-α, responses in unstimulated wells were subtracted from responses in virus-stimulated wells. Results were expressed as pg/mL.

#### ELISPOT IFN-γ

PBMC were used to evaluate the frequencies of specific IFN-γ-secreting cells (IFN-γ-SC) against the vaccine strain by ELISPOT IFN-γ as reported elsewhere [16]. The ELISPOT assay was developed using commercial mAbs (Porcine IFN-γ P2G10 and biotin P2C11, BD Biosciences Pharmingen). PBMC were stimulated with the PRRSV strain 96 V198 at a moi of 0.1; the stimulating vaccine strain had been previously titrated in a BHK-CD163 expressing cell line by end-point limiting dilution and it was adjusted to the corresponding moi just prior to conducting the test. Unstimulated and PHA-stimulated cells (10 μg/mL) were used as negative and positive controls, respectively. All tests were done in quadruplicate. Reactions were developed by adding 3-amino-9-ethylcarbazole as substrate. PRRSV-specific frequencies of IFN-γ-SC were calculated by subtracting counts of spots in unstimulated wells from counts in PRRSV-stimulated wells and were expressed as number of responding cells in 5 × 10^6^ PBMC.

### PRRSV serum neutralisation assay

Inactivated serum samples were serially diluted two-fold (1:2 to 1:4096) in 96-well plates. PRRSV strain 96 V198 vaccine virus suspension (125 μl) containing 800 CCID_50_/mL was added to each well and plates were incubated for 1 h at 36–38 °C. A BHK21-CD163 expressing cell suspension [17] containing 2.5-3 × 10^5^ cells/mL was prepared and 100 μL aliquoted to each well in a new plate. Fifty microliters of each serum-virus mix were transferred to the plate containing cells. The mixture was incubated at 36–38 °C and 5% CO_2_ for five days. A direct immunofluorescence assay technique using FITC-conjugated monoclonal antibody SDOW-17 (Rural Technologies Inc) was performed. The NA titre was determined as the inverse of the last dilution of serum that inhibited the FA signal.

### PRRSV RT-qPCR

RNA was purified from serum and nasal swab samples using the Biosprint 96 DNA Blood kit. Viraemia was measured by means of a Reverse Transcription (RT) qPCR. In brief, the purified viral RNA was used as template, reverse transcribed at 50 °C for 30 min, and denatured at 95 °C for 5 min. The PCR program of reactions consisted of 40 cycles of denaturation at 95 °C for 20 s and annealing at 53 °C for 40 s. The qRT-PCR was conducted in a 7500 Real-Time PCR System thermal cycler.

The oligonucleotide primers and dual-labeled probe used for amplification were 5’-GCACCACCTCACCCAGAC- 3′ (forward, final concentration 0.5 μM); 5’-CAGTTCCTGCGCCTTGAT- 3′ (reverse, final concentration 0.5 μM); and 5′-6-FAM-CCTCTGCTTGCAATCGATCCAGAC- BHQ1–3′ (dual-labeled probe, final concentration 0.6 μM), which correspond to base pair positions 14,792–14,809, 14,851–14,868, and 14,819–14,842, respectively, of the EU prototype strain Lelystad (Genbank accession number M96262). The amplicon consists of a 77-bp fragment from ORF7.

The genome equivalents (RNA copy number per 5 μL) were interpolated from the RNA standard curve for this assay and adjusted (RNA copy number per 1 mL of sample) according to the sample dilution.

### Data analysis

Data summaries and analyses were performed with a centralised data management system (SAS/STAT User’s Guide Version 9.3 or higher, SAS Institute, Cary, NC). Only post-challenge data (once animals were comingled) were analysed. Pre-challenge data was summarised with descriptive statistics, since the experimental unit (the pen) was not replicated. Post-challenge data was analysed, since animals had been commingled and the experimental unit was the individual animal. Prior to statistical analysis, results were transformed, where necessary, using an appropriate logarithm transformation.

For tests that had a single result after being moved into challenge housing, the transformed data were analysed using a general linear mixed model with fixed effects: treatment. For tests that had multiple results after being moved into challenge housing, the transformed data were analysed using a general linear repeated measures mixed model with fixed effects: treatment, time point, and treatment by time point interaction; and random effects: animal within treatment, which is the animal term.

Prior to statistical analysis the RT-qPCR was transformed using an appropriate logarithm transformation. The transformed data was analysed using a general linear repeated measured mixed model. Pairwise treatment comparisons were made at each time point if the treatment or treatment by time point interaction effect was significant (*P* ≤ 0.05). Treatment least squares mean and 95% confidence intervals were back-transformed for presentation. Negative samples were given a value of 50 PRRSV RNA copies/mL (1.7 log_10_ PRRSV RNA copies/mL), which corresponds to a half of the quantification limit of the technique (100 PRRSV RNA copies/mL).

Viraemia and nasal shedding were analysed with a general linear repeated measures mixed model with a logit link with fixed effects: treatment, time point, and treatment by time point interaction, and random effects: pen, block within pen, and animal within block, pen, and treatment, which is the animal term.

Linear combinations of the parameter estimates were used in a priori contrasts after testing for a significant (*P* ≤ 0.05) treatment effect or treatment by time point interaction. Comparisons were made between treatments at each time point. The 5% level of significance (*P* ≤ 0.05) was used to assess statistical differences. Least squares means (back transformed), standard errors, and 95% confidence intervals of the means were calculated for each treatment and time point. If the model did not converge, Fisher’s Exact were used for the analysis.

## Results

### Clinical observations

Eight weeks after vaccination an infectious urinary process, characterised by cystitis, urinary bladder rupture and peritonitis, occurred in the pigs allocated into the vaccinated groups (T02 and T03). One pig from T02 and two pigs from T03 died. All pigs were treated with enrofloxacin (7.5 mg/kg IM). Another pig, belonging to T02, died during the bleeding process at D56. The rest of the pigs remained healthy during the post-vaccination period. After challenge, no clinical signs associated with PRRSV infection were observed in pigs of any of the treatment groups.

### Viraemia and nasal shedding

All pigs were non-viraemic before vaccination and before challenge. After challenge, all control animals (T01) became viraemic at Day 129 (3 days after challenge), coinciding with the peak of viraemia. The length of viraemia was at least 10 days, since all control pigs were still viraemic at the end of the study. In pigs vaccinated by the IM route (T02), viraemia was detected in 8/10 pigs, and in 2 pigs it was not detected throughout the post-challenge period. The peak of viraemia was also at 3 days after challenge, although the virus load was more than 2.0 log lower than in control pigs (Table 1). At the end of the study, 10 days after challenge, all IM vaccinated pigs but one had already cleared the virus from blood. In pigs vaccinated by IN route (T03), viraemia was detected in at least one time point in all animals in the post-challenge period. The peak of viraemia was also at 3 days after challenge, although the virus load was more than 1.0 log lower than in control pigs (Table 1). At the end of the study, 10 days after challenge, all IN vaccinated pigs but two had already cleared the virus from blood.

**Table 1:** Mean (±SD) viral load in serum after challenge and percentage of viraemic pigs

	Treatment	Day of study									
		125/DC-1		129/DC + 3		132/DC + 6		134/DC + 8		136/DC + 10	
		Viral load	% viraemic pigs	Viral load	% viraemic pigs	Viral load	% viraemic pigs	Viral load	% viraemic pigs	Viral load	% viraemic pigs
Mean PRRSV viral load	T01	ND	0%	6.01 ± 0.3	100%	4.72 ± 0.1	100%	4.21 ± 0.3	100%	5.32 ± 0.8	100%
	T02	ND	0%	3.57 ± 0.6	62.5%	2.90 ± 0.5	50%	1.80 ± 0.1	12.5%	2.11 ± 0.5	12.5%
	T03	ND	0%	4.74 ± 0.7	87.5%	3.80 ± 0.4	87.5%	2.15 ± 0.3	25%	1.86 ± 0.1	25%
	Contrast										
*P* value	T01 vs T02			0.0059	NS	0.0054	NS	0.0016	0.0047	0.0087	0.0047
	T01 vs T03			NS	NS	0.0458	NS	0.0011	0.0210	0.0096	0.0210
	T02 vs T03			NS	NS	NS	NS	NS	NS	NS	NS

Values are expressed as log_10_ PRRSV RNA copies per mL of serum. *DC* day of challenge, *ND* non-detectable (below limit of detection of the technique), *NS* non-significant

Viral loads (Table 1) were significantly lower in IM vaccinated than in control pigs at days 3, 6, 8 and 10 after challenge; also, viral loads were significantly lower in IN vaccinated than in control pigs at days 6, 8 and 10 after challenge. The percentage of pigs viraemic was significantly lower at days 8 (*p* = 0.0047) and 10 (*p* = 0.0210) after challenge in both IM and IN vaccinated groups, compared to non-vaccinated pigs.

The viral load detected in nasal swabs (Table 2) was significantly lower in IM vaccinated than in control pigs at days 3 and 6 after challenge; viral loads were not significantly lower in IN vaccinated than in control pigs after challenge (Table 2). The percentage of shedding pigs was significantly lower in the IM vaccinated group, compared to the control group, at 3 days after challenge. No significant differences were detected between the IN vaccinated group and control group, or between the vaccinated groups.

**Table 2:** Mean (±SD) viral load in nasal swabs after challenge and percentage of shedder pigs

	Treatment	Day of study									
		125/DC-1		129/DC + 3		132/DC + 6		134/DC + 8		136/DC + 10	
		Viral load	% shedder pigs	Viral load	% shedder pigs	Viral load	% shedder pigs	Viral load	% shedder pigs	Viral load	% shedder pigs
Mean PRRSV viral load	T01	ND	0%	5.56 ± 0.7	100%	4.53 ± 0.5	100%	2.07 ± 0.3	20%	2.10 ± 0.4	20%
	T02	ND	0%	2.21 ± 0.6	12.5%	2.64 ± 0.4	37.5%	ND	0%	2.03 ± 0.3	25%
	T03	ND	0%	3.72 ± 0.6	62.5%	3.19 ± 0.4	62.5%	2.06 ± 0.2	25%	2.52 ± 0.3	37.5%
	Contrast										
*P* value	T01 vs T02			0.0019	0.0047	0.0119	NS	NS	NS	NS	NS
	T01 vs T03			NS	NS	NS	NS	NS	NS	NS	NS
	T02 vs T03			NS	NS	NS	NS	NS	NS	NS	NS

Values are expressed as log_10_ PRRSV RNA copies per mL. *DC* day of challenge, *ND* non-detectable (below limit of detection of the technique), *NS* non-significant

### Cell mediated immunity

#### Il-10

**Fig. 1:**
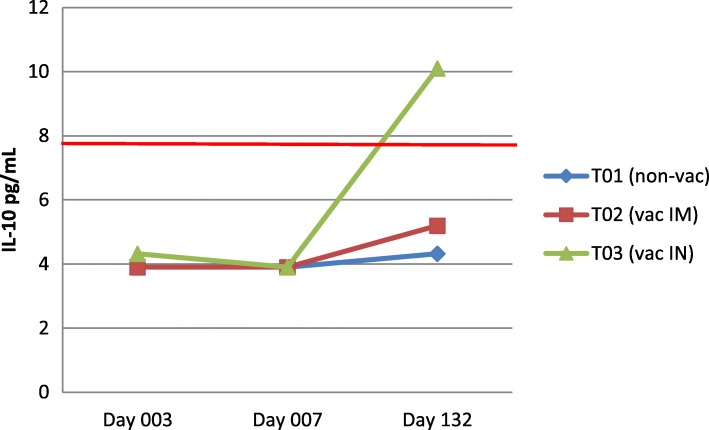
Evolution of IL-10 levels (red line indicates limit of detection: 7.8 pg/mL)

IL-10 was measured after vaccination (Days 3 and 7), and 6 days after challenge, Day 132 (Table 3, Fig. 1). Control pigs (T01) did not produce measurable IL-10 during the vaccination phase. After challenge, IL-10 was detected in one out of 5 control pigs. In pigs vaccinated by the IM route (T02), IL-10 was not produced after vaccination. After challenge, IL-10 was detected in one out of 8 pigs. In pigs vaccinated by the IN route (T03), IL-10 was produced in 1 out of 10 pigs after vaccination at Day 3. However, IL-10 was not detected in any pig at Day 7. After challenge, IL-10 was detected in 2 out of 6 tested pigs. Differences on IL-10 levels at Day 132 were not statistically significant between vaccinated and control groups, nor between vaccinated groups.

**Table 3:** Cytokine measure geometric mean (±SD) results

	Treatment	Day of study					
		3	7	28	83	125 (DC-1)	132 (DC + 6)
IL-10	T01	ND	ND				4.8 ± 1.0 (1/5)
	T02	ND	ND				5.2 ± 1.7 (1/8)
	T03	ND	ND				10.1 ± 6.4 (2/8)
*P* value	T01 vs T02						NS
	T01 vs T03						NS
	T02 vs T03						NS
IL-8	T01	ND	ND				48.1 ± 22.9 (3/5)
	T02	ND	ND				33.8 ± 12.7 (3/8)
	T03	ND	ND				69.6 ± 26.2 (6/8)
*P* value	T01 vs T02						NS
	T01 vs T03						NS
	T02 vs T03						NS
IFN-α	T01	ND	1.9 ± 0.0 (1/5)				
	T02	9.8 ± 6.1 (10/10)	32.8 ± 11.8 (10/10)				
	T03	9.5 ± 9.7 (9/10)	14.1 ± 9.9 (10/10)				
TNF-α	T01	ND	ND (1/5)^a^	ND	62.7 ± 7.6 (1/5)	ND	29.1 ± 1.5
	T02	ND	ND	ND	ND (1/8)^a^	ND	41.9 ± 1.6 (1/8)
	T03	ND	ND	ND	ND (2/8)^a^	ND	78.2 ± 2.9 (1/8)
*P* value	T01 vs T02						NS
	T01 vs T03						NS
	T02 vs T03						NS
IFN-γ	T01			1.6 ± 0.0 (5/5)	1.6 ± 0.0 (4/5)	0.0 ± 0.0 (3/4)	8.1 ± 0.5 (3/4)
	T02			22.1 ± 1.1 (9/10)	80.5 ± 8.2 (8/8)	166.5 ± 7.1 (8/8)	133.5 ± 5.7 (8/8)
	T03			51.3 ± 4.1 (8/9)	77.2 ± 8.2 (8/8)	102.2 ± 4.3 (8/8)	155.7 ± 6.6 (8/8)
*P* value	T01 vs T02					0.0003	0.0033
	T01 vs T03					0.0108	0.0011
	T02 vs T03					NS	NS

^a^Although some pigs had values above the cut-off of the technique, the mean value was below the cut-off

Results from IL-10, IL-8, IFN-α and TNF-α ELISA tests are expressed as pg/mL of serum. Results from IFN-γ ELISPOT are expressed as number of IFN-γ- secreting cells /5 × 10^5^ PBMC. In brackets, the number of positive animals per treatment and time point. *DC* day of challenge, *ND* non-detectable (below limit of detection of the technique), *NS* non-significant; blank cell indicates not measured

#### Il-8

**Fig. 2:**
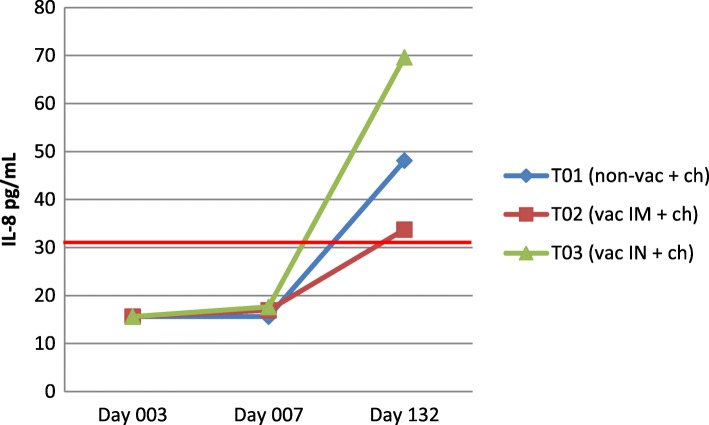
Evolution of IL-8 levels (red line indicates limit of detection: 31.25 pg/mL)

IL-8 was measured after vaccination (Days 3 and 7), and 6 days after challenge, Day 132 (Table 3, Fig. 2). Control pigs (T01) did not produce measurable IL-8 during the vaccination phase. After challenge, IL-8 was detected in 3 out of 5 control pigs. In pigs vaccinated by the IM route (T02), IL-8 was not produced after vaccination at Day 3. However, IL-8 was produced in 1 out of 10 pigs at Day 7. After challenge, IL-8 was detected in 3 out of 8 pigs. In pigs vaccinated by the IN route (T03), IL-8 was not produced after vaccination at Day 3. However, IL-8 was produced in 1 out of 10 pigs at Day 7. After challenge, IL-8 was detected in 6 out of 8 pigs. Differences on IL-8 levels at Day 132 were not statistically significant between vaccinated and control groups, nor between vaccinated groups.

#### IFN-α

**Fig. 3:**
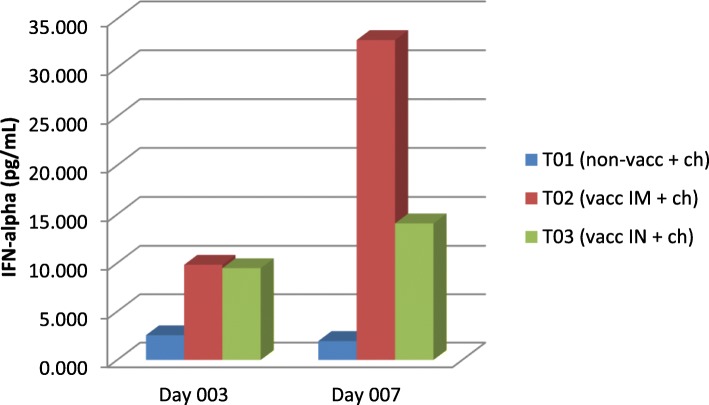
Evolution of IFN-α levels (Limit of detection: 3.9 pg/mL)

IFN-α was measured after vaccination at Days 3 and 7 (Table 3, Fig. 3). Control pigs (T01) did not produce measurable IFN-α during the vaccination phase, except in one out of 5 pigs, in which low amounts of IFN-α were detected at Day 3. At Day 7, IFN-α was not detected in any control pig. In pigs vaccinated by the IM route (T02), IFN-α was produced in all pigs at Day 3 and at Day 7. In pigs vaccinated by the IN route (T03), IFN-α was produced in 9 out of 10 pigs at Day 3, and in all pigs at Day 7.

#### TNF-α

**Fig. 4:**
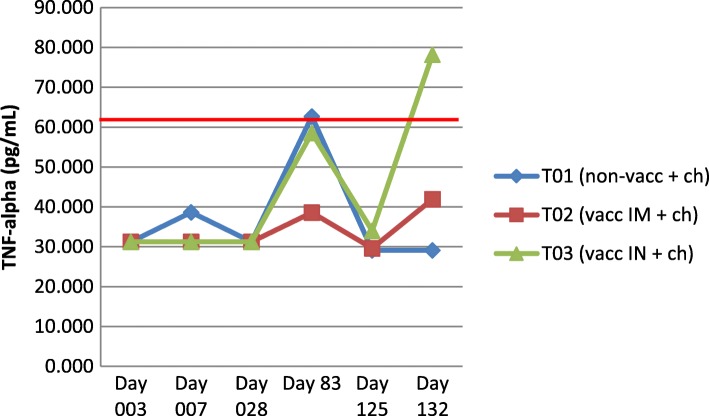
Evolution of TNF- α levels (red line indicates limit of detection: 62.5 pg/mL)

TNF-α was measured during the vaccination phase (Days 3, 7, 28, 83 and 125), and 6 days after challenge, Day 132 (Table 3, Fig. 4). Control pigs (T01) did not produce measurable TNF-α during the vaccination phase, except in one out of 5 pigs, in which low amounts of TNF-α were detected at Day 7, and another pig at Day 83. Just before challenge and after challenge, it was not detected in any pig. In pigs vaccinated by the IM route (T02), TNF-α was not produced after vaccination, except for 1 animal that produced very low amounts of TNF-α at Day 83. After challenge, TNF-α was detected in one out of 8 pigs. In pigs vaccinated by the IN route (T03), TNF-α was not produced after vaccination, except for 2 animals at Day 83. After challenge, TNF-α was detected in one out of 8 pigs. Differences on TNF-α levels at Days 125 and 132 were not statistically significant between vaccinated and control groups, nor between vaccinated groups.

#### IFN-γ

**Fig. 5:**
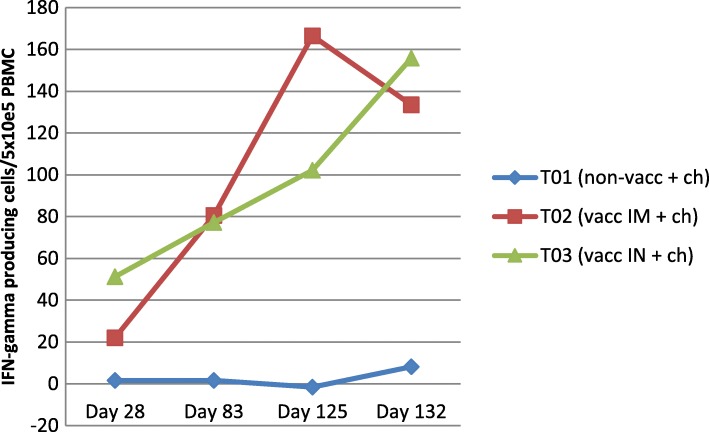
Evolution of the number of IFN-γ secreting cells, expressed as number of IFN-γ producing cells /5 × 10^5^ PBMC

IFN-γ-SC were measured during the vaccination phase (Days 28, 83 and 125), and 6 days after challenge, Day 132 (Table 3, Fig. 5). Control pigs (T01) produced very low IFN-γ secreting cells during the post-vaccination period. After challenge, there was a mild increase in the number of IFN-γ-SC. In pigs vaccinated by the IM route (T02), the number of IFN-γ-SC increased from 22 to 166 during the post-vaccination period, and were 133 after challenge. In pigs vaccinated by the IN route (T03), the number of IFN-γ-SC increased from 51 to 102 during the post-vaccination period, and to 155 after challenge. Differences in the number of IFN-γ-SC at Days 125 and 132 were statistically significant between vaccinated (both IM and IN) and control groups, but not between the vaccinated groups.

### Humoral immunity

All pigs were seronegative to PRRSV antibodies detected by ELISA before vaccination. Before challenge, at day 125, control pigs (T01) remained seronegative (mean S/P ratio 0.011), while vaccinated pigs (T02 and T03) had seroconverted. The geometric mean antibody titres were 1.681 for T02 and 1.103 for T03.

**Fig. 6:**
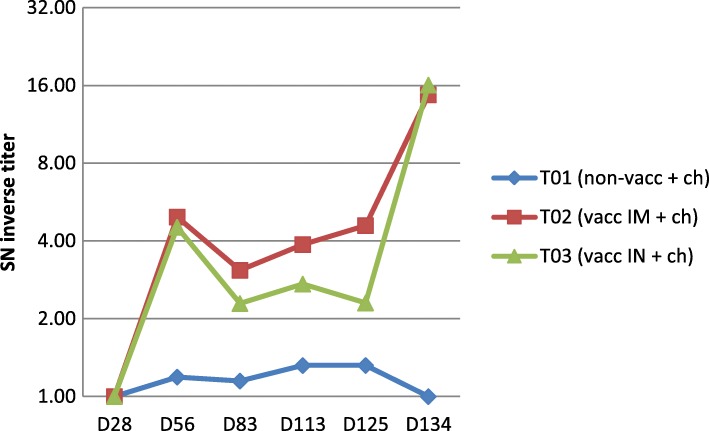
Evolution of SN titers (inverse titer). Positive ≥2

NA were measured during the vaccination phase (Days 28, 56, 83, 113 and 125), and 6 days after challenge, Day 132 (Table 4, Fig. 6). Control pigs (T01) had low to undetectable NA titres throughout the study. All vaccinated pigs developed some level of NA after vaccination and before challenge. In pigs vaccinated by the IM route (T02), NA were first detected at Day 56 after vaccination (geometric mean of 4.9), and were kept at very similar levels until challenge. After challenge, NA titres went from 4.6 (Day 125, day before challenge) to 14.7 (Day 132, 6 days after challenge). In pigs vaccinated by the IN route (T03), NA were first detected at Day 56 after vaccination (geometric mean of 4.5), and were kept at very similar levels until challenge. After challenge, NA titres went from 2.3 (Day 125, day before challenge) to 16.1 (Day 132, 6 days after challenge). Differences in NA titres at Day 132 were statistically significant between vaccinated and control groups. Differences in NA titres at Day 125 were statistically significant between pigs vaccinated by the IM route and control pigs, but not between the IN vaccinated group and the control group.

**Table 4:** Serum NA test geometric mean (±SD) results

	Treatment	Day of study					
		28	56	83	113	125 (DC-1)	132 (DC + 6)
SN antibody inverse titre	T01	ND	ND	ND	ND	ND	ND
	T02	ND	4.9 ± 3.5	3.1 ± 3.3	3.9 ± 4.0	4.6 ± 1.4	14.7 ± 4.4
	T03	ND	4.5 ± 3.9	2.3 ± 3.0	2.7 ± 2.7	2.3 ± 0.7	16.1 ± 4.8
	Contrast						
*P* value	T01 vs T02					0.0149	<.0001
	T01 vs T03					NS	<.0001
	T02 vs T03					NS	NS

Titres are expressed as the inverse of the highest dilution at which NA activity is detected. *DC* day of challenge, *ND* non-detectable (below limit of detection of the technique), *NS* non-significant

## Discussion

PRRSV is known to interact with the immune system of the host and to modulate the immune response after infection. PRRSV induces a slow and weak innate immune response [3, 4], and a delayed adaptive immune response [1, 6, 15]. The objective of this study was to characterise the development of the innate, humoral and cell mediated immune responses to PRRSV after vaccination of 1-day-old naïve pigs with a PRRSV-1 based MLV vaccine by the IM and IN routes, before and after challenge with a PRRSV-1 isolate at 18 weeks post-vaccination. Levels of cytokines IL-10, IL-8, IFN-α, TNF-α and IFN-γ, as well as levels of NA, were measured.

IL-10 is an anti-inflammatory cytokine which has been linked to regulatory T cell activity and to decreased cell-mediated immune responses, by inhibiting the induction of pro-inflammatory cytokines such as IFN-γ and TNF-α [18]. It has been hypothesised that in some strains of PRRSV the induction of IL-10 pushes the immune response towards a relatively ineffective induction of IFN-γ specific response [19]. In this study, the induction of detectable levels of IL-10 was rare, both after vaccination and after challenge. During the post-vaccination phase, a single pig (from the IN-vaccinated group) showed induction of a low level of IL-10 (10.9 pg/mL). After challenge with the Olot/91 strain, four pigs were positive for IL-10 with levels up to 346.6 pg/mL. The near absence of measurable IL-10 induction by the vaccine virus indicates that vaccination did not induce any significant response attributable to this regulatory cytokine, either in the period of innate response (day 3) or in later stages of the trial. IL-10 mediated immunomodulation/ immune dysfunction was not a general feature of this vaccine strain, as demonstrated by induction of a high number of IFN-γ-SC. This is a positive feature of the vaccine strain, since IL-10 has been related to an inhibition of the development of an effective immune response against PRRSV [10].

IL-8 is a pro-inflammatory cytokine that induces chemotaxis in target cells, primarily neutrophils and other granulocytes, causing them to migrate towards the site of infection [20]. Usually macrophages detect antigen first, and are the first cells to release IL-8 to recruit other cells. Some studies have linked an IL-8 response to enhanced resistance to PRRSV infection [21, 22]. In this study IL-8 was detected in only two pigs following vaccination, and not at all in the non-vaccinated group, suggesting that the vaccine did not generate intense inflammatory reactions. Young pigs have a reduced ability to produce IL-8 in response to an immune stimulation, compared to adults [13]. However, IL-8 was detected in the majority of vaccinated and non-vaccinated pigs after challenge, indicating that the ability of pigs to produce IL-8 was not impaired after challenge. There were no significant differences between the vaccinated and non-vaccinated groups. The lack of induction of this pro-inflammatory cytokine is consistent with the good safety profile of the vaccine (no systemic or local reactions observed after vaccination; data not shown).

TNF-α is involved in systemic inflammation and acute phase reaction as a pro-inflammatory cytokine. It is produced mainly by activated macrophages, although it can be produced by other cell types. Being an endogenous pyrogen, TNF-α is able to induce fever, apoptotic cell death, cachexia, inflammation, and to enhance viral replication [23]. In the present study, TNF-α was not detected in any vaccinated pigs before challenge. After challenge, only a minority of pigs in all three groups produced detectible levels of TNF-α, confirming the inhibitory effect of PRRSV on the induction of pro-inflammatory cytokines.

IFN-α is a type I interferon produced by infected cells, and mediates the first line of defense against viral infection (innate antiviral cytokine). Interaction of IFN-α with receptors on target cells establishes a potent antiviral state. In addition, IFN-α serves as an important link between innate and adaptive immunity, and can enhance the development of a strong T helper type 1 (Th1) inflammatory response [1]. IFN-α induction has been reported to be weak or absent for some PRRSV strains. The active inhibition of IFN-α production, and inhibition of various IFN-induced cellular gene products, has been attributed to specific viral proteins and implicated as a primary cause of the impaired immune responses to the virus [1, 24]. In this study, IFN-α was detected in all vaccinated pigs, regardless of route. IFN-α levels increased between days 3 and 7 following vaccination, while remaining low in the non-vaccinated control group. These results would be consistent with the development of antiviral responses. Consistent induction of IFN-α secretion shortly after vaccination indicates the potential for development of an effective Th1 adaptive immune response dominated by IFN-γ, which was demonstrated by frequencies of IFN-γ-SC in response to the virus well above 100 per million.

IFN-γ (type II interferon) is a pro-immune cytokine that has an ability to inhibit viral replication directly, but its most important function is immunomodulatory. IFN-γ is the primary cytokine that defines Th1 cells: Th1 cells secrete IFN-γ, as do natural killer (NK) cells. It is considered to be correlated with protection, although IFN-γ response in PRRSV infected pigs appears to be delayed [7, 25], and it is considered to be weak, compared to other swine viruses such as Aujeszky’s Disease virus [1]. In the current study, IFN-γ-SC were detected in all vaccinated pigs, increased steadily in number between days 28 and 125 post-vaccination, and remained high after challenge (means of 133 and 156 cells/5 × 10^5^ PBMC in the IM and IN treatments, respectively), confirming that vaccination can positively modulate IFN-γ gene expression and sustain IFN-γ secretion when cells are re-stimulated [7]. The number of IFN-γ-SC in non-vaccinated pigs remained low.

As mentioned, IL-8 and TNF-α are pro-inflammatory cytokines induced in some PRRSV infections. In this study IL-8 and TNF-α were either not detected, or detected in only in a minority of animals after vaccination. However, both innate and adaptive immune responses could be clearly demonstrated following vaccination by the production of IFN-α and subsequent development of IFN-γ-SC. Induction of IL-8 or TNF-α by the vaccine could have been harmful to 1-day-old pigs. The near absence of these pro-inflammatory cytokines may contribute to the observed safety of this vaccine in pre-weaning pigs.

All pigs became effectively vaccinated, as indicated by the seroconversion (by ELISA) of all pigs from both vaccinated groups. Levels of NA after vaccination were in the expected range for a PRRSV MLV vaccine. Neutralising antibodies to the vaccine strain were first detected at Day 56 in pigs vaccinated by either the IN or IM route, and were sustained at similar levels until challenge. After challenge, a boost in NA was observed in both vaccinated groups, while no evidence of a response was observed in the non-vaccinated group. Interestingly, no differences were seen for IN and IM vaccinated pigs. At challenge (Day 126) NA levels were significantly higher in IM-vaccinated pigs than in the non-vaccinated control group, and at Day 132 both IM and IN-vaccinated groups were significantly higher than the control group. In piglets, titres above 1/8 have been correlated with protection against homologous reinfection [6]. In the present study NA titres prior to challenge did not reach this value. However, it is generally acknowledged that protection against PRRSV infection is not mediated be neutralizing antibodies alone, and includes an important cell-mediated component [1]. In this study, an almost complete clearance of viraemia was achieved 10 days after challenge, in 80% of the animals vaccinated by the IM route. All these animals had detectable levels of NA and IFN-γ-SC, consistent with humoral and cell-mediated immunity both contributing to the control of infection.

Viraemia and nasal shedding results confirmed that vaccine protected pigs using both administration routes. All animals belonging to the control group were still viraemic at 10 days after challenge; in contrast, only 1/8 IM vaccinated pigs and 2/8 IN vaccinated pigs were viraemic at day 10, indicating that the immunity induced by the vaccine was able to clear the challenge virus from blood. Specifically, it was noted that those animals having a high NA titre at challenge were able to clear viraemia in 6–8 days, or even did not become viraemic. One IM-vaccinated animal with a low NA titre at challenge did not become viraemic, but had the highest IFN-γ response. NA and IFN-γ responses were measured against the vaccine strain only. Results would likely be different (lower) against a heterologous virus, such as the challenge virus used in this study. However, it is important to establish homologous baseline performance of the vaccine before considering the effects on heterologous cross-reaction on these immunoassays. The ability of the vaccine to protect against this heterologous challenge virus is evident from the viraemia results. The magnitude of the induction of NA and IFN-γ-SC (using homologous antigen) will serve as an important internal control in future studies with other challenge viruses.

Differences (other than minor) in the development of the immune response or in the efficacy of the vaccine were not observed between pigs vaccinated by the IM or IN routes. Since the mechanism of development of protection is not known for this vaccine, it was of interest to try both routes of administration. It could be speculated that both routes result in vaccine virus replication and development of a systemic immune response under these conditions.

It has been demonstrated that 1 day-old pigs can have a reduced ability to respond to immunological stimulation compared to pigs of weaning age [13]. The present study has demonstrated that piglets vaccinated at 1 day of age can develop innate, humoral and cell-mediated immune responses that are sufficient to protected them from a wild type PRRSV challenge up to at least 16 weeks after vaccination. One explanation for this apparent contradiction is the live nature of this vaccine. The vaccine virus actively replicates for weeks following vaccination, without causing disease, allowing time for the immune system of the neonatal pig to reach a greater level of maturity before eliminating the vaccine virus.

In conclusion, the administration of a PRRSV-1 based MLV to 1-day-old naïve piglets, derived from PRRS-free parent stock, was able to induce innate, cell mediated and humoral immune responses characterised by: (1) undetectable or low levels of IL-10, IL-8 and TNF-α in vaccinated pigs, (2) a mean 7-fold (IN) or 13-fold (IM) increase in IFN-α expression within the first 7 days, observed in all vaccinated pigs, (3) a gradual increase in the number of antigen-specific IFN-γ-SC, from near zero to ≥100 cells/5 × 10^5^ PBMC between days 28 post-vaccination and challenge, observed in all vaccinated pigs, and (4) induction of detectable NA in all vaccinated pigs by Day 56. After challenge with a heterologous field strain, there was a rapid boost in NA titres that was not observed in the non-vaccinated control group, indicating a priming effect of the vaccine.

## Conclusions

The administration of a PRRSV-1 based MLV to 1-day-old naïve piglets is able to induce innate, cell mediated and humoral immune responses characterised by:

- Undetectable levels of IL-10, IL-8 and TNF-α
- Seven- (IN) to 13-fold (IM) increase in IFN-α expression
- Gradual increase of IFN-γ-SC
- NA induction

After challenge with a heterologous wild type strain, a cellular immune response mediated by IFN-γ and a humoral immune response which elicited NA were able to prevent or reduce the replication of the challenge virus and allow a faster clearance of viraemia.

## Abbreviations

CCID_50_: Cell Culture Infectious Dose 50%
ELISA: Enzyme-Linked ImmunoSorbent assay
IFN-α: Interferon α
IFN-γ: Interferon γ
IL-10: Interleukin 10
IL-8: Interleukin 8
IM: Intramuscular
IN: Intranasal
mAb: Monoclonal Antibody
MLV: Modified Live Virus
MOI: Multiplicity of Infection
NA: Neutralising Antibodies
ND: Non-detectable
NK: Natural Killer
NS: Non-significant
OD: Optical Density
PBMC: Peripheral Blood Mononuclear Cells
PBS: Phosphate Buffered Saline
PHA: Phytohemagglutinin
PRRSV: Porcine Reproductive and Respiratory Syndrome Virus
RT-qPCR: Quantitative Reverse-Transcription Polymerase Chain Reaction
SC: Secreting Cells
SD: Standard Deviation
Th1: T helper type 1
TMB: 3,3′,5,5’-Tetramethylbenzidine
TNF-α: Tumor Necrosis Factor α
